# Phylogenetic diversity of stress signalling pathways in fungi

**DOI:** 10.1186/1471-2148-9-44

**Published:** 2009-02-21

**Authors:** Elissavet Nikolaou, Ino Agrafioti, Michael Stumpf, Janet Quinn, Ian Stansfield, Alistair JP Brown

**Affiliations:** 1 Aberdeen Fungal Group, School of Medical Sciences, University of Aberdeen, Institute of Medical Sciences, Foresterhill, Aberdeen, AB25 2ZD, UK; 2 Centre for Bioinformatics, Division of Molecular Biosciences, Wolfson Building, Imperial College London, South Kensington Campus, London, SW7 2AZ, UK; 3 Institute for Cell and Molecular Biosciences, Faculty of Medical Sciences, Newcastle University, 3rd Floor, Catherine Cookson Building, Framlington Place, Newcastle upon Tyne, NE2 4HH, UK

## Abstract

**Background:**

Microbes must sense environmental stresses, transduce these signals and mount protective responses to survive in hostile environments. In this study we have tested the hypothesis that fungal stress signalling pathways have evolved rapidly in a niche-specific fashion that is independent of phylogeny. To test this hypothesis we have compared the conservation of stress signalling molecules in diverse fungal species with their stress resistance. These fungi, which include ascomycetes, basidiomycetes and microsporidia, occupy highly divergent niches from saline environments to plant or mammalian hosts.

**Results:**

The fungi displayed significant variation in their resistance to osmotic (NaCl and sorbitol), oxidative (H_2_O_2 _and menadione) and cell wall stresses (Calcofluor White and Congo Red). There was no strict correlation between fungal phylogeny and stress resistance. Rather, the human pathogens tended to be more resistant to all three types of stress, an exception being the sensitivity of *Candida albicans *to the cell wall stress, Calcofluor White. In contrast, the plant pathogens were relatively sensitive to oxidative stress. The degree of conservation of osmotic, oxidative and cell wall stress signalling pathways amongst the eighteen fungal species was examined. Putative orthologues of functionally defined signalling components in *Saccharomyces cerevisiae *were identified by performing reciprocal BLASTP searches, and the percent amino acid identities of these orthologues recorded. This revealed that in general, central components of the osmotic, oxidative and cell wall stress signalling pathways are relatively well conserved, whereas the sensors lying upstream and transcriptional regulators lying downstream of these modules have diverged significantly. There was no obvious correlation between the degree of conservation of stress signalling pathways and the resistance of a particular fungus to the corresponding stress.

**Conclusion:**

Our data are consistent with the hypothesis that fungal stress signalling components have undergone rapid recent evolution to tune the stress responses in a niche-specific fashion.

## Background

Microbes require robust stress responses to survive changing environments, and in particular, pathogenic microbes must mount effective responses to counter the defences of their host. The cellular and molecular responses to stress involve both acute and adaptive phases. Acute responses generally attempt to minimise the damage caused by harmful effects of a stress, such as the immediate physico-mechanical forces imposed by an osmotic stress [1]. In contrast, adaptive responses generally promote the restoration of cellular homeostasis with a view to allowing the growth of the microbe under the new conditions [1-3]. Cells that fail to adapt adequately to a relatively severe stress may die [4,5].

To mount appropriate acute and adaptive responses, cells must sense the change in their environment and activate the cognate signal transduction pathways that induce these responses [4]. In this study we focused on hyperosmotic, oxidative and cell wall stress signalling pathways because these have been shown to contribute to fungal virulence and their sensitivity to antifungal drugs [6-11]. However, these stress signalling pathways have been characterized to the greatest extent in the relatively benign model yeast, *Saccharomyces cerevisiae*.

In *S. cerevisiae*, the HOG (High Osmolarity Glycerol) MAPK (Mitogen Activated Protein Kinase) pathway is required for responses to osmotic stress [4,12]. The Stress Activated MAP Kinase (SAPK) Hog1 is central to this pathway. Hog1 activity is regulated by the MAP kinase kinase, Pbs2 [13]. In turn Pbs2 activity is controlled by two independent osmosensing branches involving Sho1 and Sln1, respectively [14,15]. Sho1 is a putative osmosensor that regulates the Pbs2-Hog1 MAP kinase module directly [16], whereas Sln1 controls a phosphorelay system that down-regulates the MAP kinase module in the absence of hyperosmotic stress [4,17]. In response to hyperosmotic stress Pbs2 becomes activated, leading to the phosphorylation and nuclear accumulation of Hog1, and the subsequent activation of osmo-protective mechanisms such as the accumulation of the osmolyte, glycerol. A well-characterized model of the osmotic stress pathway in *S. cerevisiae *was recently described by Krantz and coworkers (2006) [18].

Oxidative stress signalling in *S. cerevisiae *has been reviewed by Moye-Rowley (2003) [19], and Ikner and Shiozaki (2005) [20]. The transcription factor Yap1p plays a key role in the activation of oxidative stress genes [21,22]. Yap1 activity is regulated by the upstream regulators Gpx3, Ybp1 and Tsa1. The heat shock transcription factor Hsf1p contributes to the activation of protective functions during the oxidative stress response [23,24]. In addition, HOG signalling is thought to regulate Yap1 synthesis. Also, the cAMP-protein kinase A signalling pathway down-regulates the activity of the partially redundant transcription factors, Msn2 and Msn4, which contribute to the core stress response that helps to protect *S. cerevisiae *against oxidative stress.

Genetic or chemical insults to the *S. cerevisiae *cell wall lead to the activation of the cell wall stress (or cell integrity) pathway, which mediates compensatory changes in cell wall architecture [25]. Cell wall stresses are detected by specific sensors in the plasma membrane, such as Wsc1, Wsc2, Wsc3, Mid2 and Mtl1 [26,27]. These sensors, together with phosphatidylinositol-4,5-biphosphate (PI4,5P2), stimulate nucleotide exchange on the GTP-binding protein Rho1 [28]. PI4,5P2 activates the guanosine nucleotide exchange factors (GEFs) Rom1/2 [29] at the plasma membrane [30]. Then, Rho1 activates the protein kinase C (Pck1) MAP kinase cascade. This cascade involves sequential activation of the MAPKKK Bck1, the MAPKK's Mkk1 and Mkk2, and the MAPK Mpk1/Slt2 [31-34].

Arguably these stress signalling pathways have been best characterised in *S. cerevisiae*. However, it is becoming clear that there are differences in stress signalling and stress sensitivities amongst fungal species, for example amongst *S. cerevisiae, Schizosaccharomyces pombe *and *Candida albicans *[35-38]. This presumably reflects their evolution in dissimilar environments where they have been exposed to different types and intensity of stress [39]. Approximately 1.5 million fungal species are thought to exist, and their great diversity reflects the heterogeneity of the niches they occupy [40]. For example, free-living ascomycetes are frequently found in the soil, tree products, plant roots and on fruit, and are often transported between substrates via insect vectors [41]. Numerous ascomycetes and basidiomycetes are important plant pathogens. Now that genome sequences are becoming available for an increasing number of diverse fungal species, it is becoming increasingly possible to perform broad bioinformatic comparisons of stress regulators across fungal species and thereby to examine the evolution of fungal stress signalling pathways. The first step in such a comparison is the identification of putative orthologues of stress signalling molecules in these fungal genomes. This approach has been used effectively to assign provisional functional annotations to protein coding genes identified by genome sequencing [42-44], to measure the effects of functional genomic variables on protein evolution rates [45-47], and applied to other areas of evolutionary genomics [48], thereby increasing our understanding of eukaryotic evolution [49,50].

The available data suggest that fungal stress signalling pathways are evolving rapidly and in a niche-specific fashion to protect different species against the contrasting environmental stresses they encounter in their diverse niches. This hypothesis implies that fungal stress resistance is evolving in a manner that is independent of fungal phylogeny. To test this hypothesis we have explored the degree of conservation of fungal stress regulators relative to their relatively well-characterised orthologues in *S. cerevisiae*, focusing on the osmotic, oxidative and cell wall stress pathways. We selected eighteen fungal species for this analysis, all of which have had their genomes sequenced and annotated. These species have evolved in divergent niches, and they show a wide variety of virulence phenotypes. The data have highlighted the strong conservation of particular fungal G-proteins and protein kinases involved in stress signalling, and the rapid evolution of upstream sensors and downstream transcription factors on these pathways. In addition we have performed the first direct comparison of the sensitivities of these fungi to osmotic, oxidative and cell wall stresses, thereby confirming the diversity of stress phenotypes amongst the species examined. Our data confirm the lack of correlation between stress sensitivity and the degree of conservation of stress regulators. Our data are consistent with the rapid polyphyletic evolution of fungal stress responses.

## Results

### Phylogenetic relationships

**Figure 1:**
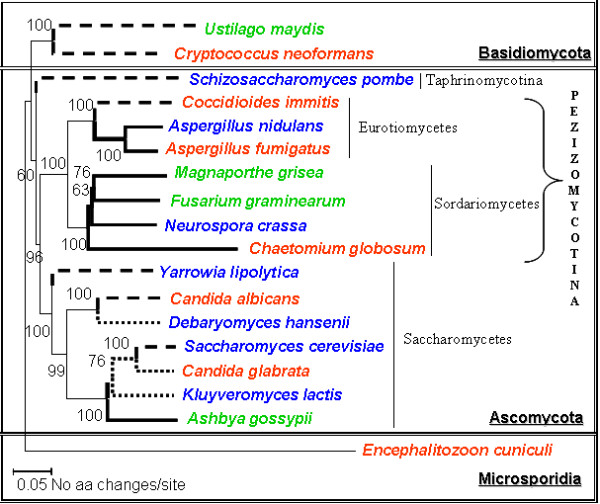
**Neighbour-joining phylogeny constructed using a concatenated alignment of 8 genes for each of the 18 fungal species**. Bootstrap scores for all the nodes are displayed. *Encephalitozoon cuniculi *was selected as an outgroup. The basidiomycetes and ascomycetes form distinct clades. Dotted lines indicate yeast-like fungi, thick straight lines indicate filamentous fungi, whereas dashed lines indicate dimorphic fungi [information adapted [144]]: red, human pathogens; green, plant pathogens; blue, benign fungi. Scale bar corresponds to 0.05 amino acid changes/site.

A preliminary objective was to reconfirm the phylogenetic relationships of the fungi under analysis (Methods). *Encephalitozoon cuniculi *was selected as outgroup for the reconstruction of the phylogenetic tree (Figure 1). As expected, the fifteen ascomycetes examined were separated into three well-resolved groups: the Saccharomycotina (7 genera, 99% bootstrap support), the Pezizomycotina (7 genera, 100% bootstrap support) and the Archiascomycetes (1 genus: *S. pombe*). The ascomycetes formed a well-supported clade, which was the sister group of the basidiomycetes. Lastly, the microsporidial species *E. cuniculi *is very different from the other two taxa, based on its genetic distance. This was entirely consistent with the recent work of Fitzpatrick and co-workers (2006) [51] who described similar phylogenies when they created a supertree based on 4,805 gene families from 42 complete fungal genomes. Therefore, our phylogenetic tree for the 18 fungal species of interest is robust and consistent with accepted views.

### Phenotypic analyses

**Figure 2:**
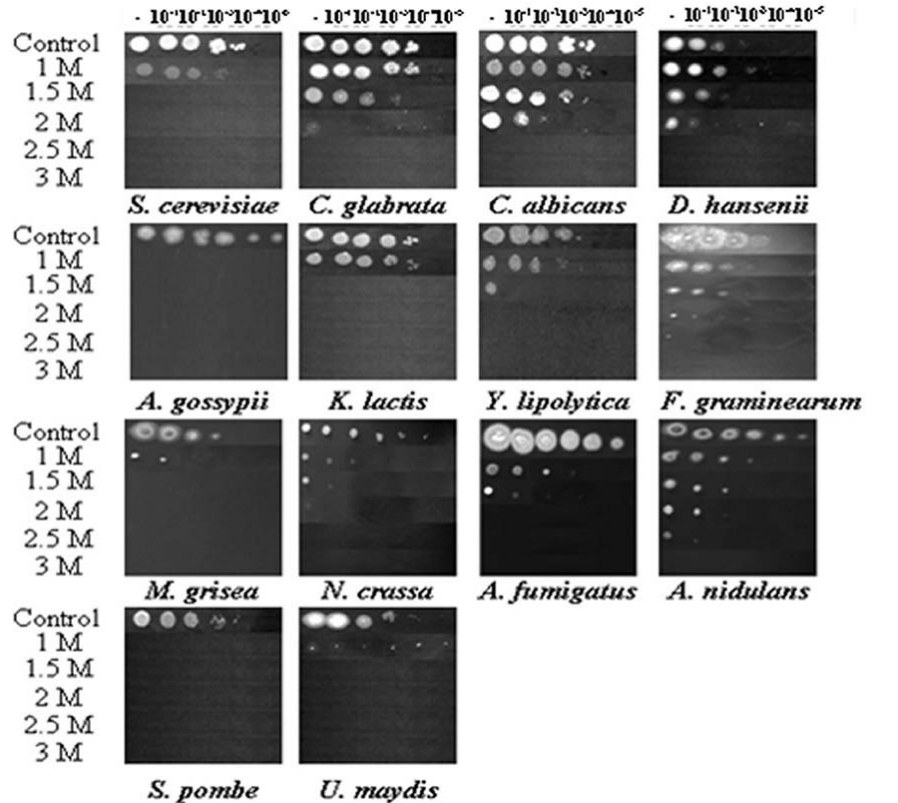
**Comparison of fungal NaCl sensitivities**. Growth of fungi on media containing various NaCl concentrations, the control plates lacking NaCl. Serial dilutions were plated as described in Materials and Methods.

Our first main objective was to compare the sensitivities of the various fungal species to osmotic, oxidative and cell wall stresses. Fourteen of the eighteen fungal species were subjected to these tests. *Cryptococcus neoformans, Coccidioides immitis, Chaetomium globosum *and *E. cuniculi *are classified as category 3 pathogens by the Advisory Committee on Dangerous Pathogens (ACDP) and hence were excluded from this part of analysis. The sensitivities of yeast-like species were examined during their exponential growth phase (OD600 = 0.8 – 1.0: data not shown). The stress sensitivities of the filamentous fungi were examined using established approaches. Hence we examined the impact of various stresses conditions upon the germination of non-vegetative *A. gossypii *and *M. grisea *spores, *Aspergillus *conidia, and *F. graminearum *and *N. crassa *macroconidia [52,53]. The data are presented in Figure 2 and Tables 1, 2, 3, 4, 5 and 6 (also see additional file 1).

**Table 1:** Relative sensitivity of fungal species to NaCl

*Relative growth (%)	NaCl [M]					
Species	Control	1.0	1.5	2.0	2.5	3.0
*S. cerevisiae*	100	75	0	0	0	0
*C. glabrata*	100	100	70	15	0	0
*C. albicans*	100	100	95	50	0	0
*D. hansenii*	100	100	71	43	0	0
*A. gossypii*	100	0	0	0	0	0
*K. lactis*	100	100	0	0	0	0
*Y. lipolytica*	100	72	22	0	0	0
*F. graminearum*	100	76	71	41	18	0
*M. grisea*	100	50	0	0	0	0
*N. crassa*	100	48	22	4	0	0
*A. fumigatus*	100	54	29	0	0	0
*A. nidulans*	100	65	52	39	22	0
*S. pombe*	100	0	0	0	0	0
*U. maydis*	100	47	0	0	0	0

* NaCl stress sensitivities were quantified by calculating the percentage growth under each condition relative to the corresponding non-stress control for that species. Examples of the NaCl plates are shown in Fig. 2.

**Table 2:** Relative sensitivity of fungal species to sorbitol

*Relative growth (%)	Sorbitol [M]			
Species	Control	1.0	2.0	3.0
*S. cerevisiae*	100	87	83	0
*C. glabrata*	100	100	96	0
*C. albicans*	100	100	100	0
*D. hansenii*	100	79	50	0
*A. gossypii*	100	50	0	0
*K. lactis*	100	96	75	0
*Y. lipolytica*	100	94	69	0
*F. graminearum*	100	75	63	0
*M. grisea*	100	75	19	0
*N. crassa*	100	65	22	0
*A. fumigatus*	100	100	71	0
*A. nidulans*	100	79	63	13
*S. pombe*	100	78	33	0
*U. maydis*	100	46	0	0

* Sorbitol stress sensitivities were quantified by calculating the percentage growth under each condition relative to the corresponding non-stress control for that species. Examples of the sorbitol plates are shown in Additional File 1.

**Table 3:** Relative sensitivity of fungal species to H_2_O_2_

*Relative growth (%)		H_2_O_2 _[mM]												
Species	Control	0.2	0.5	1.0	1.5	2.0	2.5	3.0	5.0	10	15	20	25	30
*S. cerevisiae*	100	83	83	83	75	67	58	25	0	0	0	0	0	0
*C. glabrata*	100	100	100	100	100	100	100	100	100	100	60	40	40	20
*C. albicans*	100	100	100	90	90	80	80	80	70	0	0	0	0	0
*D. hansenii*	100	82	82	82	76	53	35	24	6	0	0	0	0	0
*A. gossypii*	100	67	50	33	33	33	33	33	33	17	17	17	0	0
*K. lactis*	100	100	100	95	95	70	50	25	0	0	0	0	0	0
*Y. lipolytica*	100	83	74	83	83	70	70	70	48	0	0	0	0	0
*F. graminearum*	100	56	38	0	0	0	0	0	0	0	0	0	0	0
*M. grisea*	100	81	75	25	0	0	0	0	0	0	0	0	0	0
*N. crassa*	100	96	71	71	54	38	29	0	0	0	0	0	0	0
*A. fumigatus*	100	100	100	100	83	83	83	83	38	0	0	0	0	0
*A. nidulans*	100	83	83	83	67	67	67	50	17	0	0	0	0	0
*S. pombe*	100	100	100	100	94	81	75	50	0	0	0	0	0	0
*U. maydis*	100	65	65	65	60	60	55	35	0	0	0	0	0	0

* H_2_O_2 _stress sensitivities were quantified by calculating the percentage growth under each condition relative to the corresponding non-stress control for that species. Examples of the H_2_O_2 _plates are shown in Additional File 1.

**Table 4:** Relative sensitivity of fungal species to menadione

*Relative growth (%)		Menadione [mM]													
Species	Control	0.01	0.02	0.03	0.04	0.05	0.1	0.15	0.2	0.25	0.3	0.35	0.4	0.45	0.5
*S. cerevisiae*	100	83	83	83	83	83	83	75	33	17	17	17	17	17	17
*C. glabrata*	100	83	83	83	83	83	83	83	83	83	83	79	63	46	33
*C. albicans*	100	100	100	87	87	87	87	87	87	87	87	78	70	70	70
*D. hansenii*	100	93	93	79	57	29	0	0	0	0	0	0	0	0	0
*A. gossypii*	100	83	67	67	50	25	4	0	0	0	0	0	0	0	0
*K. lactis*	100	83	83	83	83	83	67	67	67	67	50	50	50	50	17
*Y. lipolytica*	100	83	83	67	67	67	58	54	46	17	0	0	0	0	0
*F. graminearum*	100	69	69	56	56	56	50	50	50	31	31	31	31	31	31
*M. grisea*	100	75	50	50	25	0	0	0	0	0	0	0	0	0	0
*N. crassa*	100	71	63	42	33	33	33	33	17	17	17	4	4	4	4
*A. fumigatus*	100	100	100	83	83	83	67	54	38	33	33	25	25	0	0
*A. nidulans*	100	100	100	100	100	94	88	88	75	75	75	75	50	50	50
*S. pombe*	100	100	100	100	87	80	60	47	0	0	0	0	0	0	0
*U. maydis*	100	100	81	75	56	25	25	0	0	0	0	0	0	0	0

* Menadione stress sensitivities were quantified by calculating the percentage growth under each condition relative to the corresponding non-stress control for that species. Examples of the menadione plates are shown in Additional File 1.

**Table 5:** Relative sensitivity of fungal species to Calcofluor White

*Relative growth (%)		CFW [μg/ml]								
Species	Control	20	30	50	75	100	150	200	250	300
*S. cerevisiae*	100	71	58	50	50	50	50	50	42	42
*C. glabrata*	100	100	83	83	83	83	83	83	83	83
*C. albicans*	100	83	50	17	17	0	17	17	17	17
*D. hansenii*	100	0	0	0	0	0	0	0	0	0
*A. gossypii*	100	83	67	67	67	67	67	67	67	67
*K. lactis*	100	0	0	0	0	0	0	0	0	0
*Y. lipolytica*	100	0	0	0	0	0	0	0	0	0
*F. graminearum*	100	87	80	80	80	80	80	80	80	73
*M. grisea*	100	100	75	75	75	50	50	50	50	50
*N. crassa*	100	75	75	75	75	71	71	71	71	71
*A. fumigatus*	100	100	83	83	83	83	50	50	50	50
*A. nidulans*	100	83	83	83	83	83	83	54	54	38
*S. pombe*	100	94	94	94	94	81	81	81	81	81
*U. maydis*	100	46	46	38	38	23	8	8	8	8

* CFW stress sensitivities were quantified by calculating the percentage growth under each condition relative to the corresponding non-stress control for that species. Examples of the Calcofluor White plates are shown in Additional File 1.

**Table 6:** Relative sensitivity of fungal species to Congo Red

*Relative growth (%)		CR [μg/ml]								
Species	Control	20	50	100	150	200	250	300	400	500
*S. cerevisiae*	100	95	85	75	55	40	40	25	20	20
*C. glabrata*	100	83	83	83	83	83	83	83	83	67
*C. albicans*	100	83	67	67	67	67	67	67	63	17
*D. hansenii*	100	31	0	0	0	0	0	0	0	0
*A. gossypii*	100	67	50	50	50	50	50	50	50	50
*K. lactis*	100	100	100	95	95	95	80	80	80	80
*Y. lipolytica*	100	25	0	0	0	0	0	0	0	0
*F. graminearum*	100	94	69	56	44	44	44	44	44	44
*M. grisea*	100	94	75	75	75	75	75	75	75	50
*N. crassa*	100	75	75	71	71	71	71	71	71	71
*A. fumigatus*	100	100	100	100	100	100	83	50	33	33
*A. nidulans*	100	79	71	71	71	71	71	71	71	71
*S. pombe*	100	100	100	100	100	100	100	100	100	100
*U. maydis*	100	25	0	0	0	0	0	0	0	0

* CR stress sensitivities were quantified by calculating the percentage growth under each condition relative to the corresponding non-stress control for that species. Examples of the Congo Red plates are shown in Additional File 1.

### Osmotic stress sensitivity

The sensitivity of each fungal species to osmotic stress was examined using a range of NaCl and sorbitol concentrations, which impose ionic and non-ionic osmotic stresses, respectively [54]. *S. pombe *and *A. gossypii *exhibited the greatest sensitivity to NaCl, their growth being completely inhibited by 1 M NaCl. In contrast, *C. albicans, C. glabrata *and *D. hansenii *were the most resistant to NaCl (Figure 2, Table 1). While *C. albicans *and *D. hansenii *are members of the CTG clade (*i.e*. the clade of organisms in which the CTG codon is decoded as serine instead of leucine), *C. glabrata *is not (Figure 1). The growth of these species was only slightly inhibited by 1.5 M NaCl, and no growth was observed at concentrations above 2 M NaCl. The rest of ascomycetes showed relatively low resistance to NaCl. Of the filamentous fungi, *F. graminearum *and *A. nidulans *showed the highest resistance to NaCl. The human pathogen, *A. fumigatus *was more sensitive to NaCl, and the plant pathogen, *U. maydis *was most sensitive to this stress.

Similar observations were made when sorbitol was used to impose osmotic stress (Table 2, see also additional file 1). Once again *S. pombe *and *A. gossypii *were the most sensitive to sorbitol, and *C. albicans *and *C. glabrata *were amongst the most resistant species. Interestingly, compared with other species, the halotolerant yeast *D. hansenii *was relatively sensitive to sorbitol but resistant to NaCl (Tables 1 and 2). Also the human pathogen, *A. fumigatus *was more sensitive to NaCl than sorbitol, by comparison with the other species. Indeed all of the human pathogens tested were resistant to sorbitol. With the exception of *A. gossypii, M. grisae *and *N. crassa*, the ascomycetes tested were relatively resistant to sorbitol (Table 2). The basidiomycetes species tested (*U. maydis*) was sensitive to both osmotic stresses.

### Oxidative stress sensitivity

The sensitivities of the fungi to oxidative stresses were tested by plating on media containing hydrogen peroxide (H_2_O_2_) and menadione (Tables 3 and 4; see also additional file 1). *F. graminearum *and *M. grisae *were particularly sensitive to H_2_O_2 _whereas the human pathogen *C. glabrata *was exceptionally resistant to this oxidative stress. *M. grisea *was also sensitive to menadione as well as H_2_O_2_, suggesting that this plant pathogen is sensitive to oxidative stresses in general. In contrast *F. graminearum, C. albicans *and *K. lactis *were relatively resistant to menadione. It is interesting to note that, in general, the human pathogens were relatively resistant to the oxidative stresses tested, whereas the plant pathogens tested were relatively sensitive.

### Cell wall stress sensitivity

Finally, we tested the sensitivity of the cell wall stresses using Calcofluor White and Congo Red. These inhibitors disturb cell wall biosynthesis in *S. cerevisiae *and *C. albicans*, activating compensatory changes in cell wall architecture via the cell wall stress signalling (or cell integrity) pathway. The majority of fungi investigated were resistant to both Calcofluor White and Congo Red (Tables 5 and 6). *S. pombe *and *C. glabrata *stood out as the most resistant species to these cell wall stresses, whereas *D. hansenii *and *Y. lipolytica *were the most sensitive to these stresses. In contrast, *K. lactis *and *C. albicans *were sensitive to Calcofluor White, but relatively resistant to Congo Red. The basidiomycete *U. maydis *was sensitive to both inhibitors. Regarding the other plant pathogens, *F. graminearum *was relatively resistant to Calcofluor White, but sensitive to Congo Red. The reverse was true for *M. grisae*. In general the human pathogens were relatively resistant to both stresses, the exception being the sensitivity of *C. albicans *to Calcofluor White. The non-pathogenic *Pezizomycotina *were resistant to Calcofluor White and Congo Red.

### Conservation of stress signalling modules

Having tested the sensitivity of the fungal species to osmotic, oxidative and cell wall stresses, we examined the degree of conservation of regulatory proteins on the corresponding stress signalling pathways. *C. neoformans, C. immitis, C. globosum *and *E. cuniculi *were included in these analyses. We assumed for the purposes of this study that the functions of orthologues are conserved across the fungal species examined. This allowed us to map putative regulators to the corresponding stress signalling pathways that have been characterized in *S. cerevisiae*. However, this assumption does not always hold, and the possibility that these regulators might execute alternative functions in a particular species should be borne in mind.

**Figure 3:**
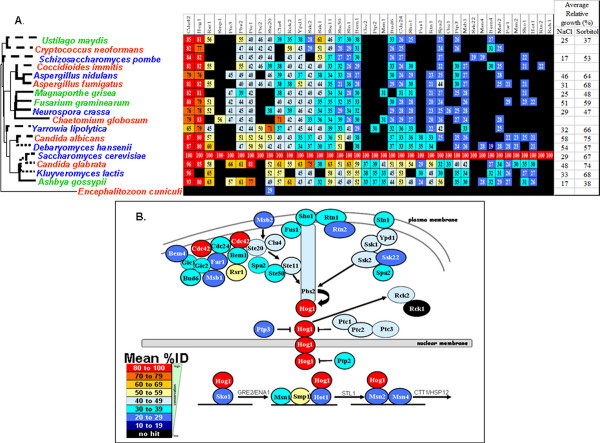
**Degree of conservation of fungal osmotic stress regulators**. (A) Orthologues of *S. cerevisiae *osmotic stress regulators in the fungi analysed. The organisms are ordered according to their position in the phylogeny, and the regulators ordered according to their mean %ID across all of the fungal species examined. Closed indicates no orthologue identified. (B) Mean conservation (%ID) of osmotic stress regulators across the fungal species examined based on the model of the osmotic stress pathway in *S. cerevisiae *described by Krantz and co-workers (2006) [18].

**Figure 4:**
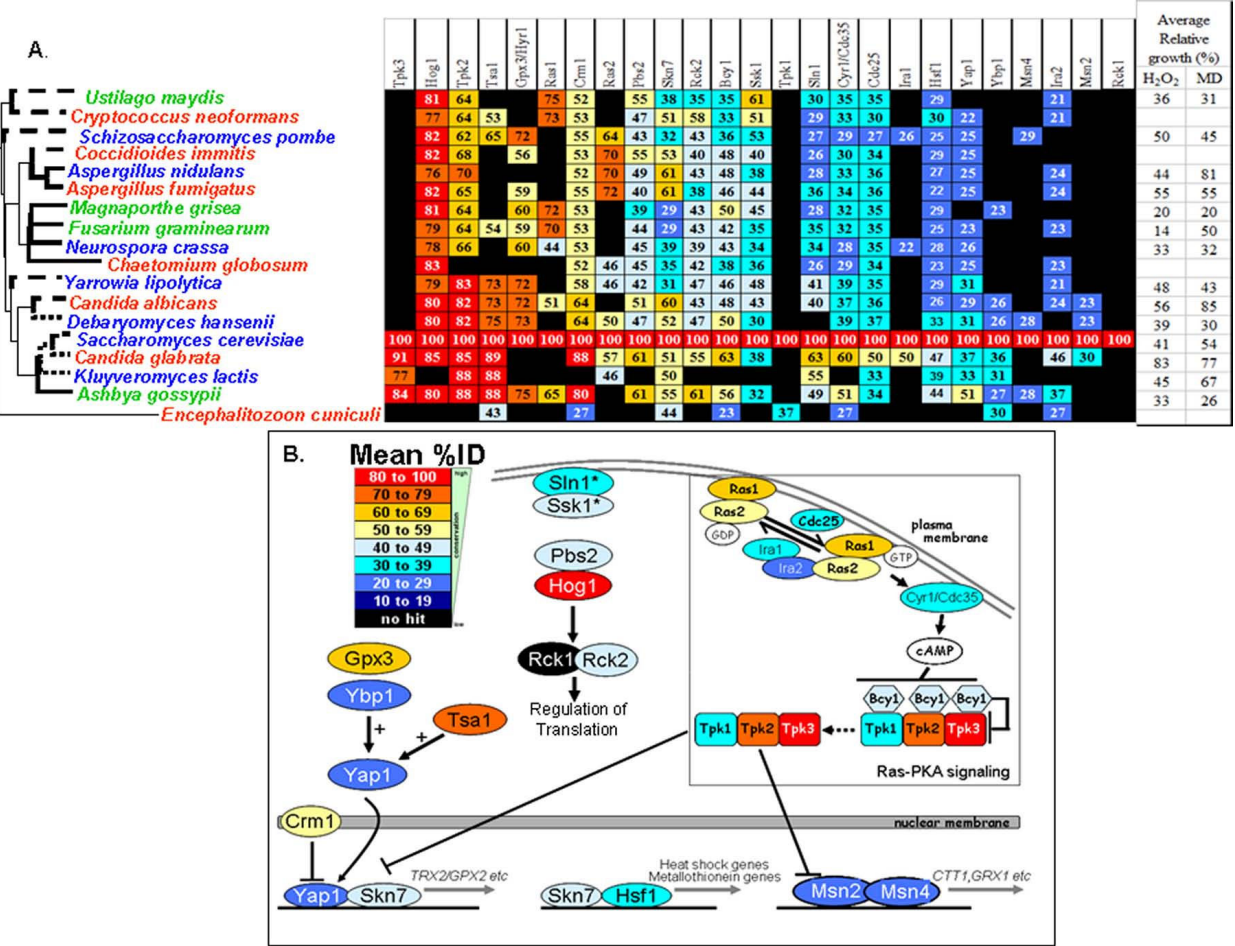
**Degree of conservation of fungal oxidative stress regulators**. (A) Orthologues of *S. cerevisiae *oxidative stress regulators in the fungi analysed. As before, the fungi are ordered according to their position in the phylogeny, and the regulators ordered according to their mean %ID. Closed indicates no orthologue identified. (B) Mean conservation (%ID) of oxidative stress regulators across the fungal species examined based on the *S. cerevisiae *oxidative stress pathway adapted from reviews by Moye-Rowley (2003) [19] and Ikner and Shiozaki (2005) [20].

**Figure 5:**
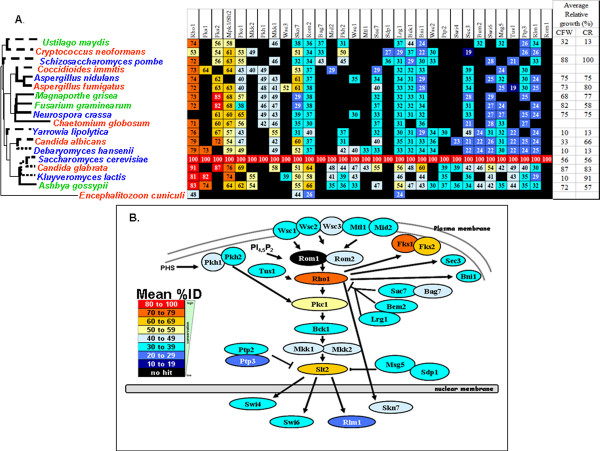
**Degree of conservation of fungal cell wall stress regulators**. (A) Orthologues of *S. cerevisiae *cell wall stress regulators in the fungi analysed. As before, the fungi are ordered according to their position in the phylogeny, and the regulators ordered according to their mean %ID. Closed indicates no orthologue identified. (B) Mean conservation (%ID) of cell wall stress regulators across the fungal species examined based on the *S. cerevisiae *cell wall stress pathway adapted from Levin (2005) [25].

First, putative orthologues were identified for each *S. cerevisiae *protein in the other seventeen fungal species by screening for reciprocal best hits, as described in Materials and Methods. Lists of fungal orthologues are presented as additional file 2. Proteins involved in osmotic, oxidative and cell wall stress signalling were then selected, based on recent models of these pathways in *S. cerevisiae*. We used the model of the osmotic stress signalling pathway reported by Krantz and co-workers (2006) [18], the cell wall stress pathway described by Levin (2005) [25], and a model of the oxidative stress signalling pathway based on the reviews of Moye-Rowley (2003) [19] and Ikner and Shiozaki (2005) [20]. We then examined the conservation of each signalling pathway in each fungus by collating the percent identities for the relevant regulators, relative to their *S. cerevisiae *orthologues, as reported by BLASTP (Figures 3, 4 and 5). The orthologues are listed in the additional file 2.

Mean percent identities were then calculated for each regulator. This allowed the comparison of osmotic, oxidative and cell wall stress signalling components across the eighteen fungal species under investigation (Figures 3, 4 and 5). The Hog1 SAPK and the G-protein Cdc42 stand out as being the most highly conserved signalling molecules in the osmotic stress pathway (Figure 3). Other central components of the MAP kinase module (Ste11, Pbs2) and phosphorelay module (Ypd1, Ssk1, Ssk2) are reasonably well conserved across the diverse species analysed. However, the sensors and transcriptional regulators that lie upstream and downstream of these models are generally poorly conserved.

With regard to oxidative stress signalling (Figure 4), members of the glutaredoxin and thioredoxin systems are well conserved (Tsa1 and Gpx3). Also, Ras (Ras1/2) and protein kinase A (Tpk1/2/3) are well conserved. (The strong conservation of Hog1 has already been mentioned.) Once again, downstream transcription factors involved in oxidative stress signalling are poorly conserved. This is the case for Yap1 orthologues, even though they are known to play key roles in the oxidative stress response in *S. cerevisiae, S. pombe, C. albicans *and *C. glabrata *[7,21,22,55]. This also holds for Msn2/4 orthologues. However in this case, while Msn2/4 orthologues contribute to oxidative stress responses in *S. cerevisiae *and *C. glabrata*, they do not do so in *C. albicans *[36,55,56].

Signalling components on the cell wall stress pathway also show great diversity with respect to their degree of evolutionary conservation (Figure 5). Once again some core components are strongly conserved most notably a G-protein (Rho1), protein kinase C (Pkc1), and a MAP kinase (Slt2). Also, the sensors of cell wall stresses (Wsc1/2/3, Mtl1, Mid2) are less well conserved than these central signalling components. Also, the transcription factors that are downstream targets of these signalling modules are less well conserved (Swi4/6, Rlm1). However, subunits of the glucan synthase are highly conserved (Fks1/2). These lie downstream of Rho1 signalling, and are essential for cell wall biosynthesis [25,57,58].

Individual diagrams have been created to illustrate the degree of conservation of each regulator in each fungus relative to *S. cerevisiae *(see additional file 3). Generally, the above observations hold across the individual fungal species we examined. In general core signalling molecules are more highly conserved than upstream and downstream components.

## Discussion

In this study we have compared directly the stress sensitivities of a diverse group of fungal species for the first time (Figure 1). These species were selected on the basis that their genomes had been sequenced and annotated (Table 7). This allowed us to examine the evolutionary conservation of stress signalling components amongst ascomycete, basidiomycete and microsporidial species. Our study has focused on osmotic, oxidative and cell wall stress signalling. A number of significant observations have been made.

**Table 7:** Strains and data sources

Organisms	Strains	Ecologic Niche	References
*A. gossypii*	ATCC10895	Cotton	[145]
*A. fumigatus*	Af293	Decaying organic & plant material	[146]
*A. nidulans*	FGSCA4	Tropical & subtropical regions	[147]
*C. albicans*	SC5314	Skin, mucosa	[148]
*C. glabrata*	CBS138	Mouth, gastrointestinal tracks	[149]
C. globosum*	CBS148.51	Soil, air and plant debris	Unpublished
C. immitis*	RS	Soil (dessert like areas of southwest USA)	Unpublished
C. neoformans*	JEC21	Soil contaminated by pigeon droppings	[150]
*D. hansenii*	CBS767	All types of cheese, dairies, brines	[149]
E. cuniculi*	GB-M1	Urine, blood, kidney	[151]
*F. graminearum*	PH-1	Cotton, wheat, barley, bean, soybean	Unpublished
*K. lactis*	NRRLY-1140	Milk and milk products	[149]
*M. grisea*	Guy-11	Rice	[152]
*N. crassa*	OR74A	Dead plant matter after fibres	[153]
*S. cerevisiae*	S288C	Oak tress (oils), surface of fruits	[154]
*S. pombe*	927C	Grapes (wine fermentation)	[155]
*U. maydis*	521	Soil plant material, maize (corn), grasses	Unpublished
*Y. lipolytica*	CLIB122	Oil fields, cheese, sausages	[149]

* C. globosum, *C. immitis*, *C. neoformans *and *E. cuniculi *are classified as category 3 pathogens by ACDP (Advisory Committee on Dangerous Pathogens) and hence were used only for bioinformatics analysis in current study. NCBI GI numbers are provided for *E. cuniculi*.

Genome sequences sources; *A. gossypii*: ; *A. fumigatus *and *C. neoformans*: ; *A. nidulans*, *C. globosum*, *C. immitis*, *F. graminearum*, *M. grisea*, *N. crassa *and *U. maydis *; *C. albicans *; *C. glabrata*, *D. hansenii*, *K. lactis *and *Y. lipolytica *; *S. cerevisiae *; *S. pombe *; *E. cuniculi *

The first main observation was that the fungal species examined displayed wide variation in their resistance to osmotic, oxidative and cell wall stresses (Tables 1, 2, 3, 4, 5 and 6). For example, while some fungi showed acute sensitivity to osmotic stress (*S. pombe, A. gossypii*), others were relatively resistant (*C. albicans, F. graminearum*). Similarly, some fungi were relatively sensitive to oxidative stress (*D. hansenii, M. grisea*), whereas others were extremely resistant to this type of environmental insult (*C. glabrata*).

Interestingly, some species showed differential sensitivities to the alternative osmotic, oxidative and cell wall stresses. For example, *D. hansenii *was resistant to NaCl, but less resistant to sorbitol (Tables 1 and 2). NaCl imposes a salt (ionic) stress in addition to an osmotic stress, whereas sorbitol imposes a non-ionic stress [54,59]. *D. hansenii *has been isolated from saline environments such as sea water [60] and concentrated brines [61]. This species is known to be more osmotolerant than *S. cerevisiae *[60], accumulating glycerol and to a lesser extent arabitol as compatible solutes [62,63]. This difference is due in part to more effective sodium extrusion by *D. hansenii *[64].

*A. nidulans*, *F. graminearum, C. albicans, K. lactis *and *S. cerevisiae *showed differential responses to the oxidative stresses tested (Tables 3 and 4). These fungi were more resistant to menadione than H_2_O_2_. This was consistent with the findings of Mutoh and co-authors (2005) [65] who previously reported that *S. pombe *is more sensitive to H_2_O_2 _than menadione. This would appear to suggest that these fungi are better able to detoxify the superoxide generated by menadione, than the peroxide anions generated by H_2_O_2_. However in *S. cerevisiae*, peroxide is generated from superoxide by superoxide dismutases, this peroxide subsequently being detoxified by catalases [66,67]. Therefore it might seem counterintuitive for some fungi to be more resistant to menadione. However when transition metals are present, H_2_O_2 _can also be converted to the hydroxyl radical, which is more potent than the superoxide radical [67,68]. Therefore the relative sensitivity of some fungi to H_2_O_2 _might reflect a reduced capacity to detoxify hydroxyl radicals.

Differential sensitivities to the cell wall stress were also observed (Tables 5 and 6). Calcofluor White and Congo Red responses interact with different components in the fungal cell wall. Calcofluor White binds to nascent chitin, inhibiting the assembly of chitin chains in the wall [69-72]. In contrast, Congo Red is generally thought to inhibit β-1,3-glucan assembly in the cell wall [73-76]. *S. cerevisiae *mutants with an increased chitin content in the cell wall are more sensitive to Calcofluor White, whereas mutants with reduced chitin are more resistant to Calcofluor White [76-78]. It follows that the differential fungal sensitivities to Calcofluor White and Congo Red might be explained, at least in part, by the different chitin and β-glucan contents of their cell walls. In *S. cerevisiae*, *C. albicans *and *S. pombe*, β-1,3-glucan accounts for 50 – 55% of the cell wall dry weight and is responsible for much of the mechanical strength of the cell wall [58,79,80]. Chitin is a relatively minor constituent of the yeast cell wall comprising 1 to 2% of the cell wall dry weight in *S. cerevisiae*, *C. albicans *[58,81,82]. The *S. pombe *cell wall was reported to contain no chitin [83-85], but more recently a small amount of chitin was detected [0.3% of dry weight: [86]]. This low chitin content probably accounts for the Calcofuor White resistance of *S. pombe *(Table 5). However the filamentous fungi were relatively resistant to Calcofuor White and Congo Red, and yet chitin is a major component of their cell walls [82,87]. The *Neurospora *cell wall contains 10–20% chitin [87-89], whereas in *A. nidulans *chitin constitutes up to 40% of the cell wall [90]. Therefore additional mechanisms must account for the relative Calcofuor White resistance of the filamentous fungi.

Our second main observation was that there was no clear correlation between fungal phylogeny and stress resistance (Figures 3, 4 and 5). In some cases closely related fungi displayed similar stress sensitivities. For example, the *Aspergillus *species examined (Eurotiomycetes) were relatively resistant to the cell wall stresses (Calcofluor White and Congo Red: Figure 5) and displayed similar responses to osmotic stresses (NaCl and sorbitol: Figure 3). The Saccharomycetes, *C. albicans *and *D. hansenii*, which also belong to the CTG clade (where the CTG codon is translated as serine, rather than leucine), were both highly resistant to NaCl (Figure 3). However in other cases, closely related fungal species displayed contrasting stress sensitivities. For example, *C. glabrata *was much more resistant to osmotic, oxidative and cell wall stresses than *S. cerevisiae *(Figures 3, 4 and 5). *F. graminearum *was more resistant to NaCl and menadione than *M. grisae*. Also, *C. albicans *was more resistant to H_2_O_2_, menadione and Congo Red than *D. hansenii*.

This was extended by more systematic analysis of individual stress response pathways. The mean percent identity was calculated for all components of a given stress response pathway in each species. These data were then plotted against the differential impact of that same stress upon *S. cerevisiae *and the comparator species (by calculating the difference between their mean growth inhibitions for the stress in question). If pathway sequence divergence is indicative of increasing differences in stress response, then a negative correlation would be expected of this analysis (i.e. a lower mean pathway percentage identity would correlate with greater differences in stress response). In fact in every case, the regression R^2 ^coefficients were less than 0.09 (completely non-significant), and for all but one stress, correlations were weakly positive (see also additional file 4). Therefore, this analysis confirmed that there is no correlation between the degree of conservation stress regulators and the similarity of stress phenotypes. We conclude that fungal stress phenotypes have evolved rapidly and in a polyphyletic manner.

Presumably this rapid evolution of fungal stress phenotypes has been driven by local and niche-specific environmental pressures. If this was the case, one might expect to observe a correlation between the stress phenotype of a particular fungus and the nature of the environmental niche that it occupies. Our data are consistent with this view. For example as described above, *D. hansenii*, which was has been isolated from saline environments, is resistant to salt stress but less resistant to a non-ionic osmotic stress (Figure 3). Also, the human pathogens we examined (*A. fumigatus*, *C. glabrata *and *C. albicans*) were highly resistant to oxidative stress, whereas the plant pathogens (*M. grisea*, *F. graminearum*, *A. gossypii *and *U. maydis*) were sensitive to this type of stress (Figure 4).

Phagocytic cells are a first line of defence against fungal infections, generating superoxide, H_2_O_2_, and hydroxyl radicals in an attempt to destroy the phagocytosed pathogen [91-93]. The major fungal pathogen *C. albicans *activates oxidative stress responses when exposed to human blood, macrophages or neutrophils [11,94-97]. Indeed the virulence of *C. albicans *is dependent upon its ability to counteract oxidative stress [6,8,10,11]. Therefore it is hardly surprising that *C. albicans *has evolved to become relatively resistant to oxidative stress [98]. The same is true for another opportunistic fungal pathogen. *C. glabrata *is highly resistant to oxidative stress [55,92,99-101] even though phylogenetically it is more closely related to *S. cerevisiae *than to *C. albicans *[102].

Our third main observation was that, despite the critical importance of stress responses for the environmental robustness of the fungi, the upstream sensors and downstream transcriptional regulators on three stress signalling pathways show a low degree of sequence conservation (Figures 3, 4 and 5). Core components of the stress pathways are relatively strongly conserved. In some cases these core components play multiple cellular roles. For example, core components of the cell wall stress (cell integrity) pathway and in Ras-cAMP-protein kinase A signalling play key roles in the regulation of growth and cell polarity [30,103,104]. Therefore their strong sequence conservation is to be expected. In contrast, the upstream sensors and downstream transcriptional regulators generally play more specific roles in the detection of environmental stress and the activation of stress-specific responses. Clearly the evolutionary divergence of specific sensors or transcriptional regulators could contribute to the differential stress phenotypes of these fungal species by modulating the sensitivity of each species to a particular type of stress and tuning the strength of the molecular response to that stress. Therefore, the low degree of conservation of the upstream and downstream signalling components is entirely consistent with the rapid polyphyletic evolution of fungal stress resistance in response to niche-specific selection pressures.

Our evolutionary comparison of stress signalling components was based on the identification of the fungal orthologues of *S. cerevisiae*. Orthologues were defined on a genome-wide basis by identifying the best reciprocal hits. In some cases it was not possible to identify orthologues in all of the species examined. In many of these cases this probably reflects the lack of a genuine orthologue. However in some cases genuine orthologues might have fallen below the BLAST cut-off due to their low level of sequence similarity. In other cases the presence of an orthologue was not detected because of the existence of closely related paralogous gene pairs in *S. cerevisiae *that arose through the ancient genome duplication [105]. In these cases, the BLAST search for *S. cerevisiae *'paralogue A' identified a particular fungal 'gene X', but the reciprocal BLAST search for 'gene X' identified *S. cerevisiae *'paralogue B', thereby yielding no reciprocal best hit. Despite these health warnings, most commonly used orthology resources are based on reciprocal best hits. These include Clusters of Orthologues Genes [C/KOGs: [106,107]], INPARANOID [108,109] and the NCBI resource, HomoloGENE [110]. Also, it should be noted that, as our comparisons were based on *S. cerevisiae*, fungal proteins that exist in other species but not in *S. cerevisiae *will not have been identified in this study. Nevertheless, our study has provided a comprehensive list of fungal orthologues to *S. cerevisiae *proteins in seventeen divergent fungal species (see additional file 2). We have used this resource to study fungal stress signalling, but it is freely available for the analysis of other aspects of fungal molecular and cell biology.

## Conclusion

Our comparison of the stress resistance of diverse fungal species has revealed a high degree of variation in their resistance to osmotic, oxidative and cell wall stresses. Fungal species that are closely related in phylogenetic terms did not necessarily display similar levels of stress resistance. Human pathogens tended to be more resistant to stress, with the exception of *Candida albicans *which was relatively sensitive to the cell wall stress, Calcofluor White. Plant pathogens tended to be sensitive to oxidative stress.

We have examined the degree of conservation of osmotic, oxidative and cell wall stress signal transduction pathways in the eighteen diverse fungal species. Central components of these signalling pathways are generally well conserved, whereas upstream sensors and downstream transcriptional regulators have diverged to a greater extent. No correlation between the degree of conservation of stress signalling pathways and the resistance of a particular fungus to the corresponding stress was observed. The data reinforce the view that stress signalling components have evolved rapidly to protect fungal species against the environmental insults they experience in their specialized niches.

## Methods

### Strains and growth media

The strains used in this study are summarized in Table 7. *C. albicans*, *Candida glabrata*, *Debaryomyces hansenii*, *Kluyveromyces lactis*, *S. cerevisiae*, *S. pombe*, *Ustilago maydis *and *Yarrowia lipolytica *were grown in YPD [111,112]. *Ashbya gossypii *mycelia or spores were grown on AFM [*Ashbya *full medium: [113,114]]. *Aspergillus fumigatus *mycelia or conidia were grown on PDA [potato dextrose agar: [115]]. *Aspergillus nidulans *mycelia or conidia were grown on MNVUU (minimal medium: [116,117]). *Fusarium graminearum *mycelia or macroconidia were grown on SNA [synthetic nutrient poor agar: [118,119]]. *Magnaporthe grisea *mycelia or spores were grown on CM [complete medium: [120]]. *Neurospora crassa *mycelia were grown on solid Vogel's medium using D-glucose instead of sucrose as described by Vogel (1956) [121] and Selitrennikoff and Sachs (1991) [122]. All strains were maintained as frozen stocks and then cultured on the appropriate media.

Using the above methods we were able to standardize the growth conditions under which the stress phenotypes for yeast and filamentous species were examined as far as was practically possible. However, these growth conditions were not optimal for some species, and this might have affected their stress resistance.

### Growth conditions

*C. albicans*, *C. glabrata*, *D. hansenii*, *K. lactis*, *S. cerevisiae*, *S. pombe*, *U. maydis *and *Y. lipolytica *colonies were picked from YPD plates, inoculated into 5 ml of YPD, and incubated overnight at 30°C at 200 rpm [112].

Fragments of *A. gossypii *mycelia were placed on AFM agar plates and incubated for 7 days at 30°C [adapted from [123]]. Mycelial mats were then removed, resuspended in 800 μl H2O and 200 μl zymolyase-100T, and incubated at 37°C for 4 hours. 1 ml of 0.03% Triton X-100 were added and the spores were collected by centrifugation at 5000 rpm for 5 min. The spores were washed twice with 0.03% Triton X-100.

Disks of *A. fumigatus *mycelia (2.5 cm diameter) were inoculated on PDA plate and incubated upside down at 37°C for 5 days [115]. The conidia harvested by gentle scraping three times into 3 ml 0.1% Tween 20, and the conidial suspension filtered through 4 layers of Miracloth (Calbiochem, Merck Biosciences, Nottingham, UK) to remove hyphae.

Disks of *A. nidulans *mycelia (2.5 cm diameter) were inoculated on MNVUU plates and incubated upside down at 28°C for 7 days [117]. Conidia were then harvested using the same procedure as for *A. fumigatus *but water was used instead of Tween solution to scrape the plate.

Small fragments of agar containing *F. graminearum *mycelia were placed on fresh SNA plates [119]. Plates were sealed with Parafilm and incubated under blue/white light for 8–10 days at 25°C. Macroconidia were harvested using the same procedure as for *A. nidulans*.

Filter papers containing *M. grisea *mycelia were placed on CM plates and incubated upside down at 25°C for 14 days [adapted from [120]]. Spores were harvested using the same procedure as for *A. nidulans*.

*N. crassa *mycelia were grown in 250 ml conical flasks containing 40 ml solid VgS medium [adapted from [124]]. Flasks were incubated in the dark at 28°C for 3 days, and then in the light for 2 more days. Macroconidia were harvested into 50 ml dH2O, and the suspension transferred to a 15 ml Falcon tube. This was repeated three times to maximize the yield of macroconidia.

### Stress sensitivity assays

Osmotic stress was applied using NaCl (0–3 M range) and Dsorbitol (0–3 M range). Oxidative stress was imposed using H2O2 (0–30 mM range) and menadione sodium bisulfate (0–0.5 mM range). Cell wall stress was applied using Calcofluor White (0–300 μg/ml range) and Congo Red (0–500 μg/ml range).

Overnight cultures of *C. albicans*, *C. glabrata, D. hansenii*, *K. lactis*, *S. cerevisiae*, *S. pombe*, *U. maydis *and *Y. lipolytica *grown in YPD at 30°C, were used to inoculate 10 ml of YPD to a starting OD600 of 0.1. The cells were grown at 30°C at 200 rpm to an OD600 of 0.8 – 1.0. These exponential cells were then serially diluted and 3 μl drops of each dilution (10^0^–10^-5^) were spotted onto YPD plates containing the appropriate stress treatment. Growth was assessed after 2 days incubation at 30°C.

In general, the stress assays for the filamentous fungi were performed under the growth conditions described above. Exceptions were the media used for *A. fumigatus *and *N. crassa *conidial stress assays. *A. fumigatus *conidia were grown on YG [yeast extract agar: [125]]. For *N. crassa *L-sorbose was added to Vogel's growth medium to promote colonial growth [126]. Fresh spore or conidial suspensions were serially diluted and 3 μl drops of each dilution (10^0^–10^-5^) were spotted onto plates containing the appropriate stress treatment. Growth of the filamentous fungi was examined after 2 days incubation, except for *M. grisea*, which was examined after 5 days incubation. Experiments were repeated at least three times.

### Quantitative analysis of stress resistance

To semi-quantitatively compare the stress resistances of the fungal species analysed under the conditions tested, the percentage of growth of each species was calculated relative to their non-stress control for each stress condition. For each species, the total number of spots observed across the dilutions (10^0^–10^-5^) for each stress condition tested was counted and expressed as percentages of those on the corresponding control plates (Tables 1, 2, 3, 4, 5 and 6). To obtain a global view of the response of each fungal species to each osmotic (NaCl and sorbitol), oxidative (H_2_O_2 _and MD) and cell wall (CFW and CR) stress the mean relative growth (%) was calculated for each species under analysis for each condition tested. To measure relative growth, the amount of growth in the presence of stress was divided by the amount of growth observed for unstressed cells of the same species and expressed as a percentage.

### Phylogenetic analyses

Eight *S. cerevisiae *proteins were used as queries for our phylogenetic analyses: actin [127], 3- phosphoglycerate kinase [128], translation elongation factor EF-1 alpha [129], the cyclin-dependent protein kinase, Cdc28 [130], adenylate cyclase [131,132], and the transcription factors Gcn4, Mig1 and Fap1 [133-135], which belong to different gene families. These *S. cerevisiae *protein sequences were retrieved from the *Saccharomyces *Genome Database (SGD: ). The sequences of the orthologues of these proteins in the other fungal species under analysis were retrieved by BLASTP [136] using the SGD protein sequences as queries (see additional file 5). Manual searches were undertaken at the National Center for Biotechnology Information using NCBI databases, including the non-redundant protein sequence database (nr database) currently containing approximately 900,000 sequences . BLASTP search parameters were set to default. Phylogenetic analyses were performed using MEGA3.1 [137] available at . Sequences were aligned using ClustalW [138]. A concatenated phylogenetic tree was then produced by neighbour-joining (NJ) clustering [139]. The phylogeny was drawn using the p-distances method to correct for multiple amino acid substitutions per site and rate heterogeneity amongst sites. The substitution of amino sites per site was 0.05 (scale bar underneath tree). Clade stability was assessed using 1000 bootstrap replicates. Phylogenetic trees were presented using TreeView [140].

### Identification of putative orthologues

The complete annotated fungal genome sequences were retrieved from the databases in Table 7[144-155]. Putative orthologues were identified for each *S. cerevisiae *protein in each fungal species under analysis using the reciprocal best hit (rbh) method [46,141]. We define 'putative orthologues' as two proteins, one from each fungal genome, that are each other's reciprocal best hit [142]. Perl scripts were used to reformat the amino acid sequence data (formatdb), to perform reciprocal BLASTP searches, and to generate output files that provide the accession number for each orthologue, its percentage identity to the corresponding *S. cerevisiae *protein and the match score. Automated reciprocal BLASTP searches were performed [143] using the default parameters except that scoring parameter compositional adjustments were set to "no adjustment" and the filter parameter was set automatically to "low complexity regions". Putative orthologues were not identified for all *S. cerevisiae *proteins in every species above the default BLASTP cut-off, which was set to 10 by default. Only the reciprocal best hits identified in this way were considered for further analysis. Where no significant reciprocal hit was identified, the score was left blank.

## Authors' contributions

EN carried out the phenotypic and bioinformatic analyses and drafted the manuscript. IA created the PERL program for the automated BLASTP searches under the supervision of MS. JQ participated in the oxidative stress analysis. IS participated in the bioinformatic analysis and helped to draft the manuscript. AJPB conceived of the study, participated in its design and coordination, and prepared the last version of the manuscript. All authors read and approved the final manuscript.

## Supplementary Material

Additional file 1 **Sensitivity of fungi to different stresses.** Fungal stress sensitivity data: (A) sorbitol; (B) H_2_O_2_; (C) enadione sodium bisulfite; (D) Calcofluor White; (E) Congo Red. Click here for file

Additional file 2 **List of fungal orthologues.** Lists of reciprocal best hits: (A) complete list of all fungal orthologues; (B) osmotic stress signalling orthologues; (C) oxidative stress signalling orthologues; (D) cell wall cell stress signalling orthologues. Click here for file

Additional file 3 **Conservation of fungal osmotic, oxidative and cell wall stress pathways.** Figures illustrating the degree of conservation of signalling molecules on stress pathways in each of the fungal species examined: (A) osmotic stress signalling pathway; (B) oxidative stress signalling pathway; (C) cell wall cell stress signalling pathway. Click here for file

Additional file 4 **No correlation exists between fungal stress phenotypes and the degree of conservation of fungal stress regulators.** Plot showing no significant correlation between the degree of conservation of oxidative stress regulators and the resistance of the fungal species to oxidative stress. Click here for file

Additional file 5 **Proteins used for phylogenetic analysis.** Details of fungal orthologues used to construc the phylogenetic tree. Click here for file
