# Supplementary material for: Phylogenetic diversity of stress signalling pathways in fungi
Source: BMC Evol Biol. 2009 Feb 21;9:44. doi: 10.1186/1471-2148-9-44 (PMC2666651; doi:10.1186/1471-2148-9-44)
Supplement: Additional file 1 — Sensitivity of fungi to different stresses. Fungal stress sensitivity data: (A) sorbitol; (B) H2O2; (C) enadione sodium bisulfite; (D) Calcofluor White; (E) Congo Red. [file 1471-2148-9-44-S1.pdf]

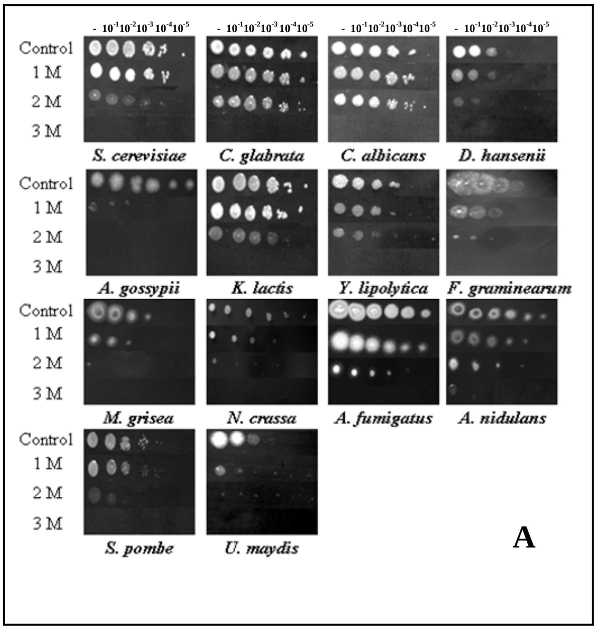

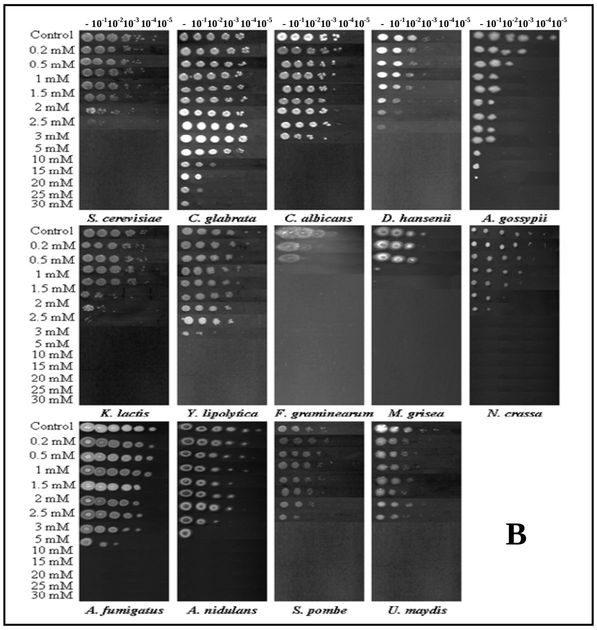

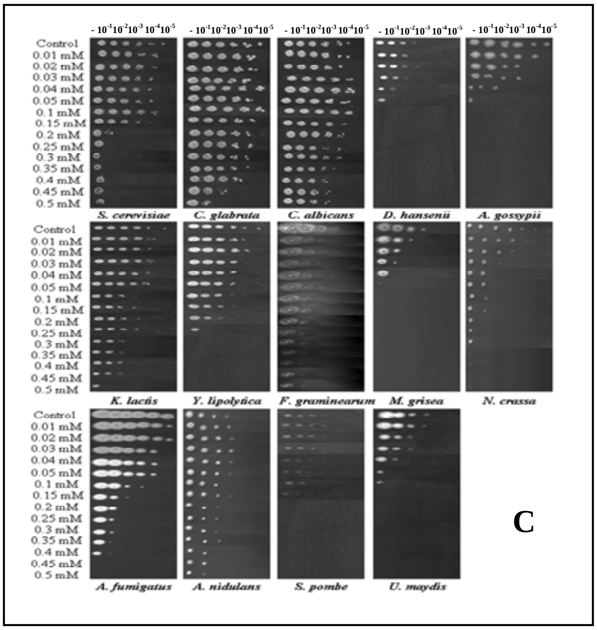

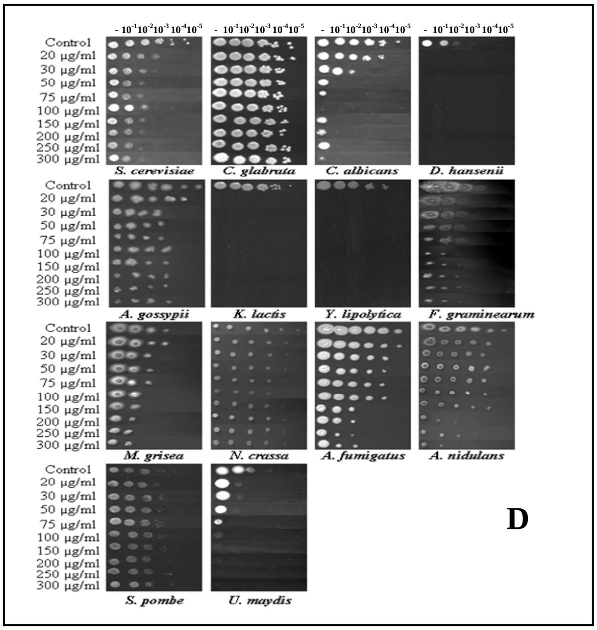

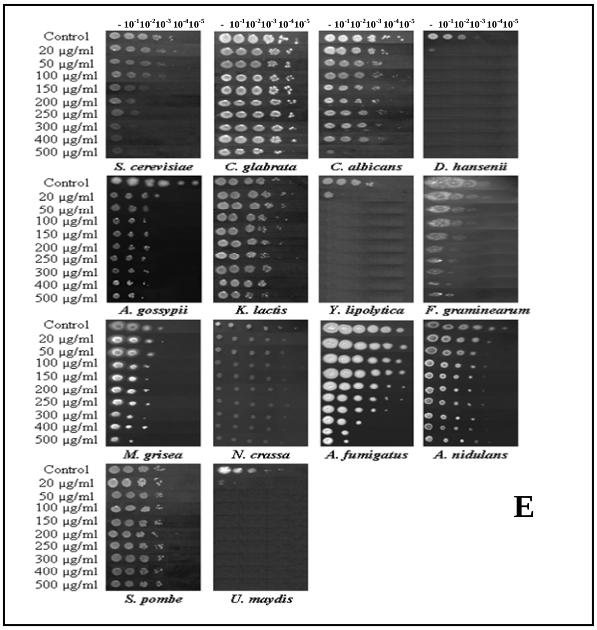

Growth of species under analysis on media plates containing (A) 1, 2 and 3 M sorbitol (B) 0.2, 0.5, 1, 1.5, 2, 2.5, 3, 5, 10, 15, 20, 25, 30 mM H_2O_2 (C) 0.01, 0.2, 0.03, 0.04, 0.05, 0.1, 0.15, 0.2, 0.25, 0.3, 0.35, 0.4, 0.45, 0.5 mM menadione (D) 20, 30, 50, 75, 100, 150, 200, 250, 300 µg/ml Calcofluor White (E) 20, 50, 100, 150, 200, 250, 300, 400, 500 µg/ml Congo Red . Growth was also assessed on control plates lacking an inhibitor (first row of each picture).
