# Supplementary figures and images for: Phylogenetic diversity of stress signalling pathways in fungi
Source: BMC Evol Biol. 2009 Feb 21;9:44. doi: 10.1186/1471-2148-9-44 (PMC2666651; doi:10.1186/1471-2148-9-44)

# A Osmotic stress pathway

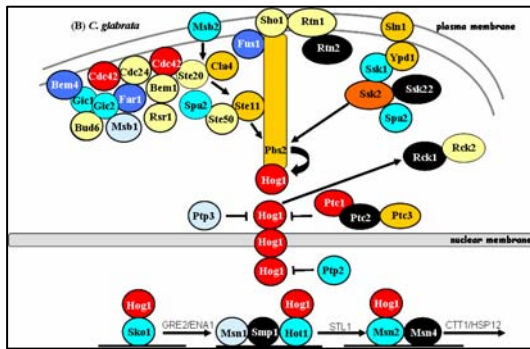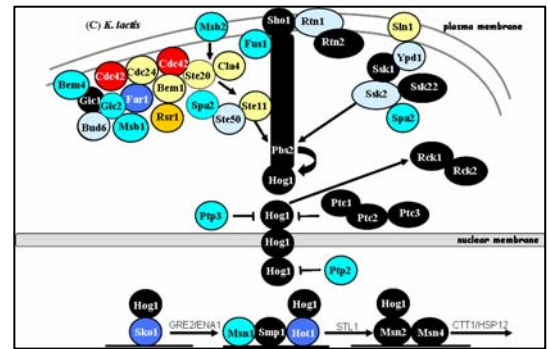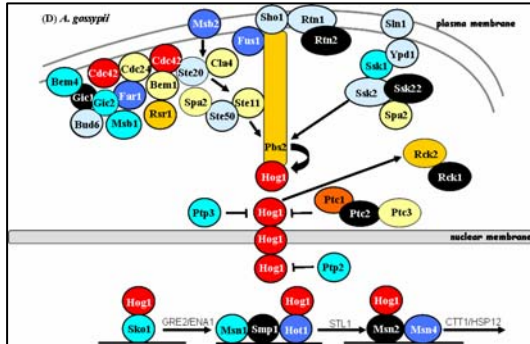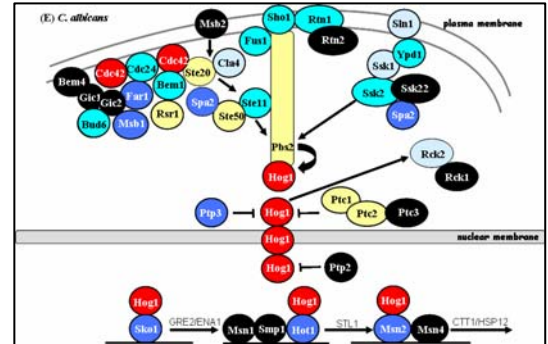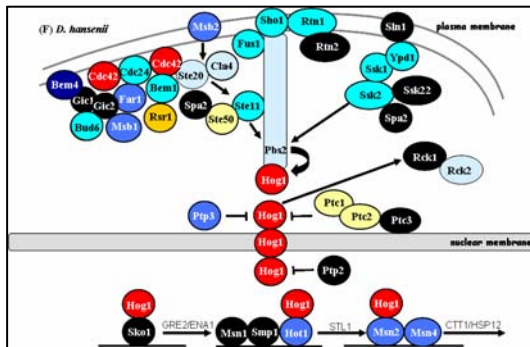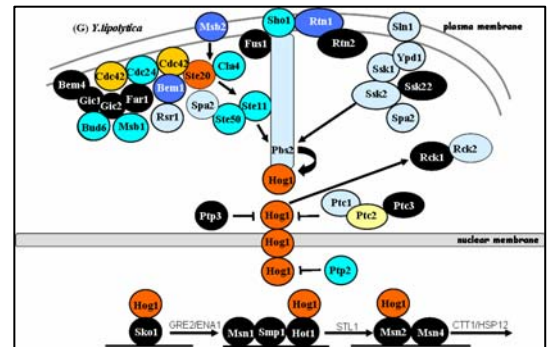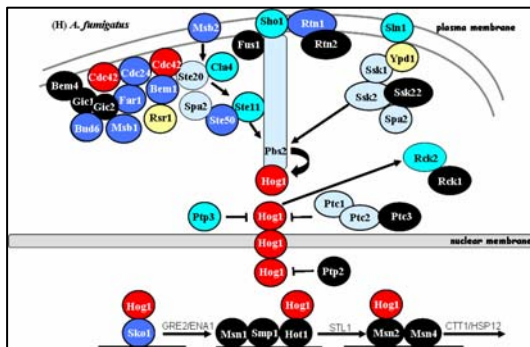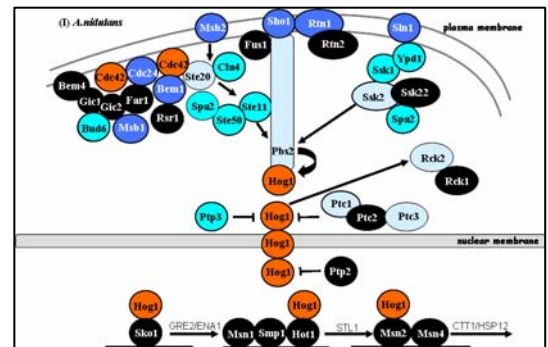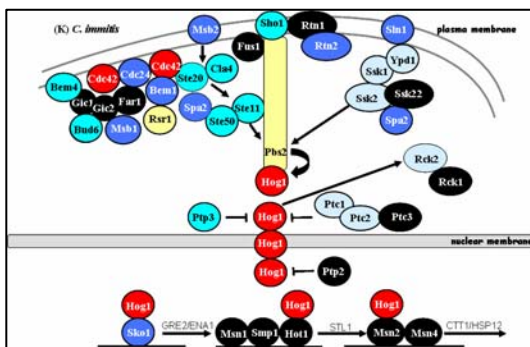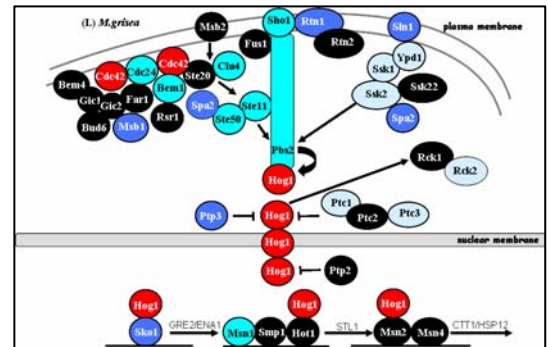

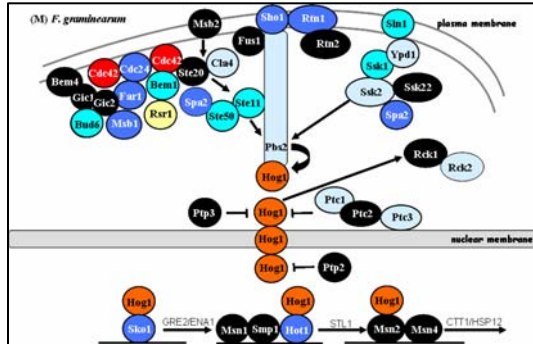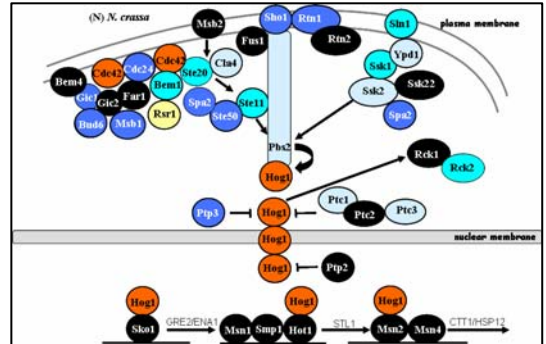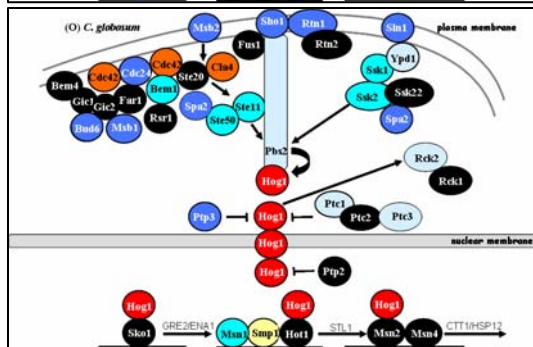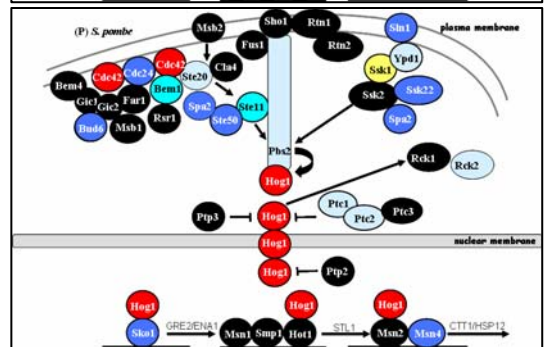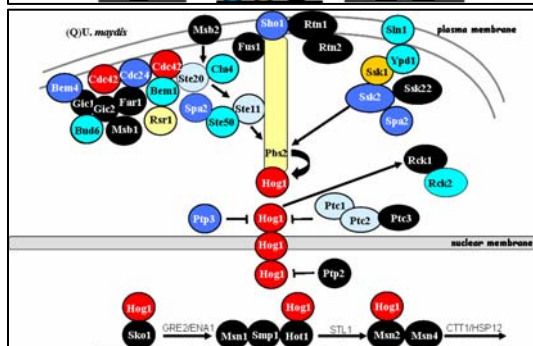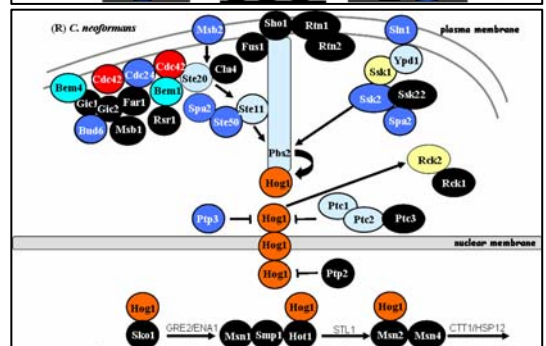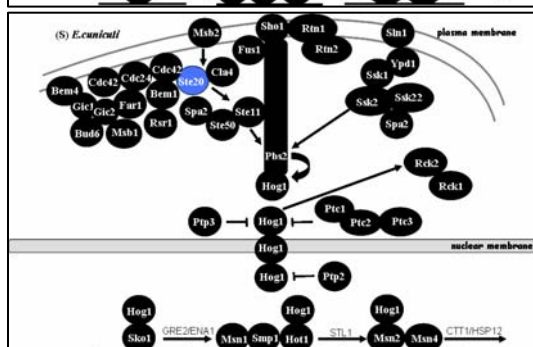

## B Oxidative stress pathway

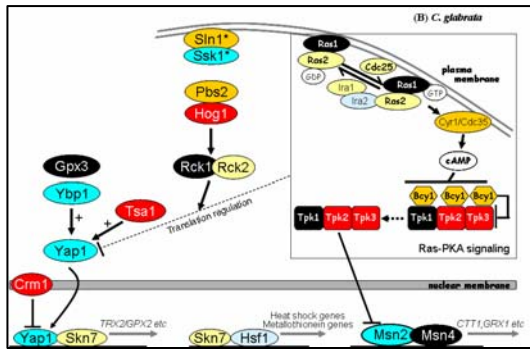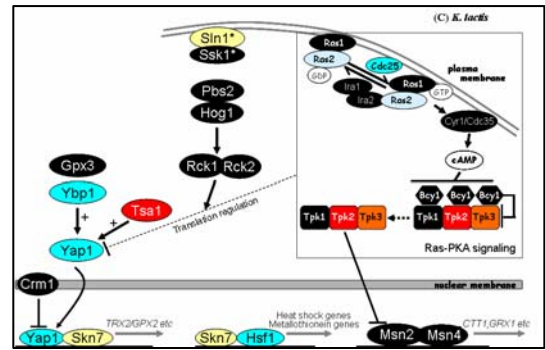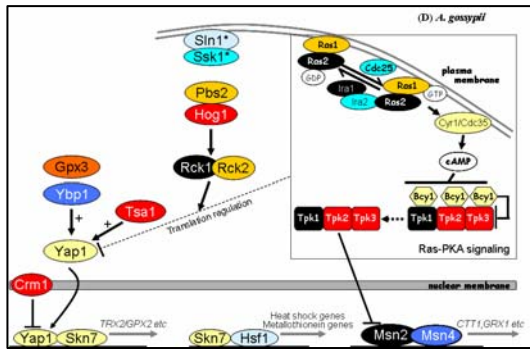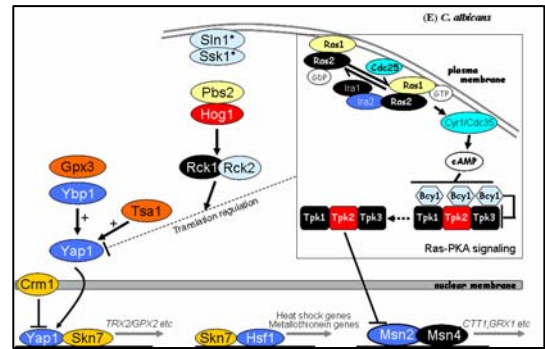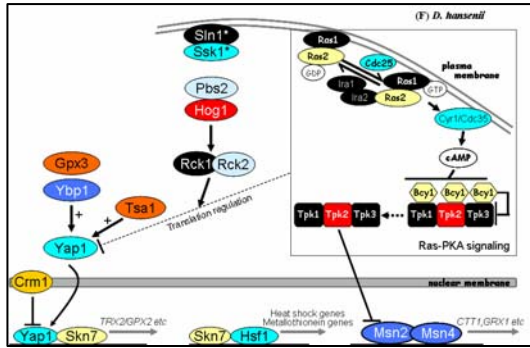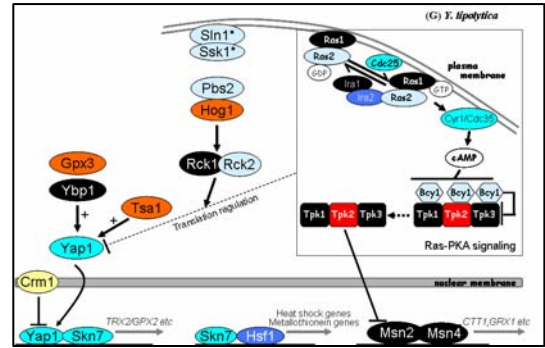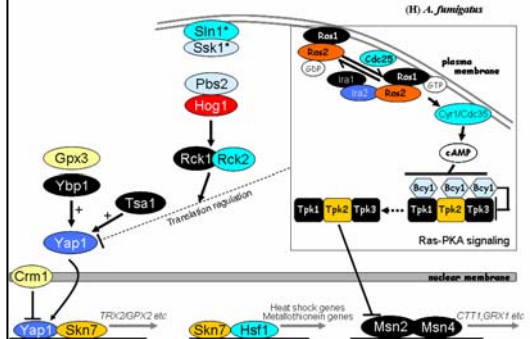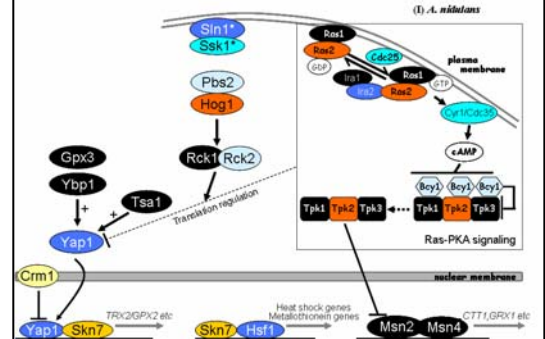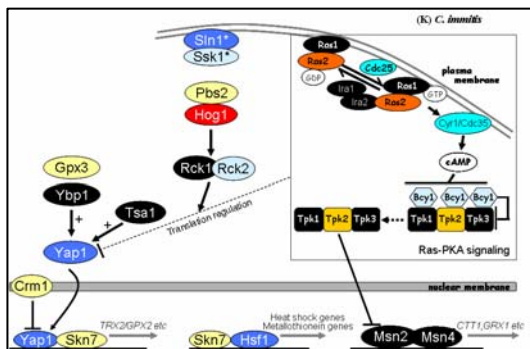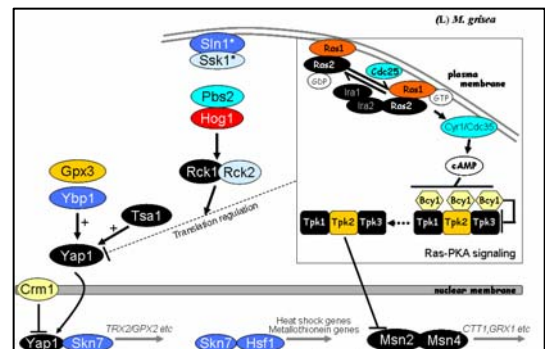



# C Cell wall stress pathway

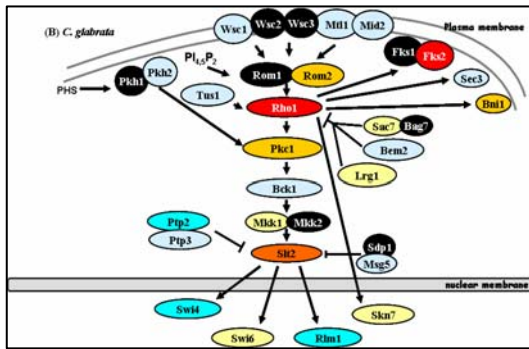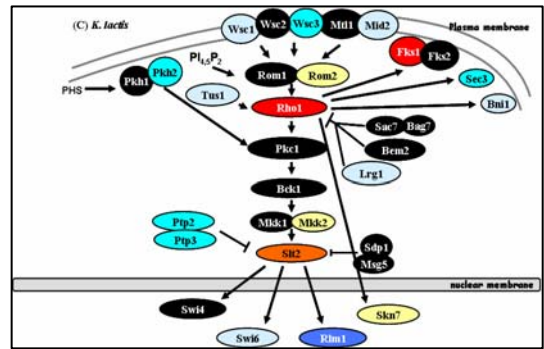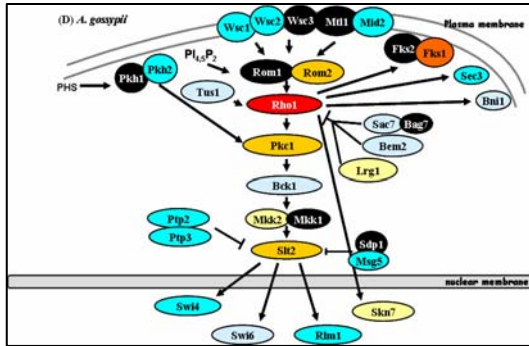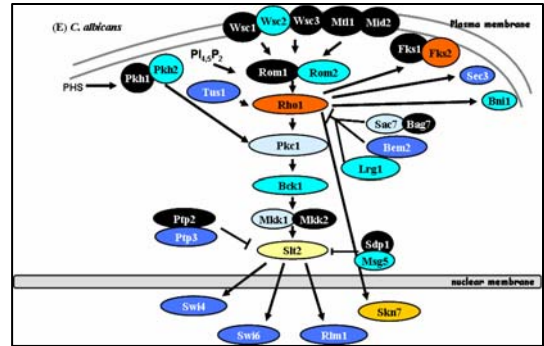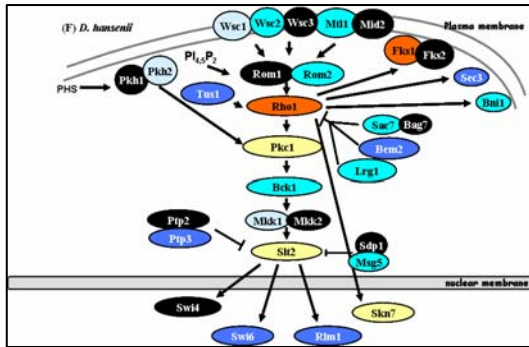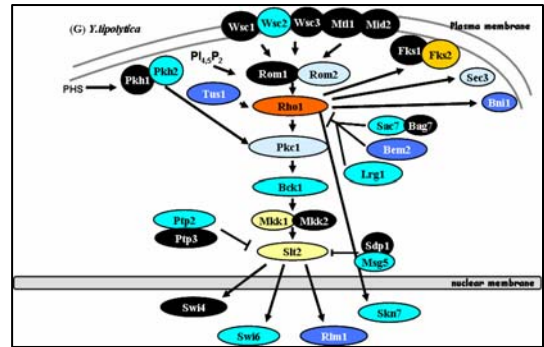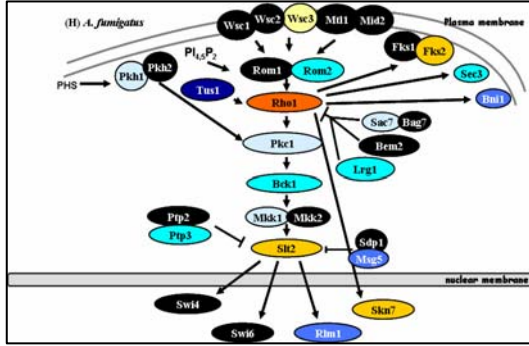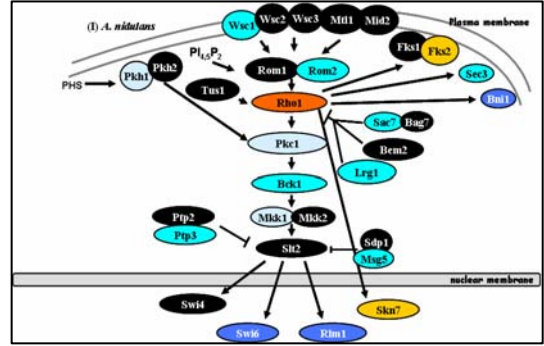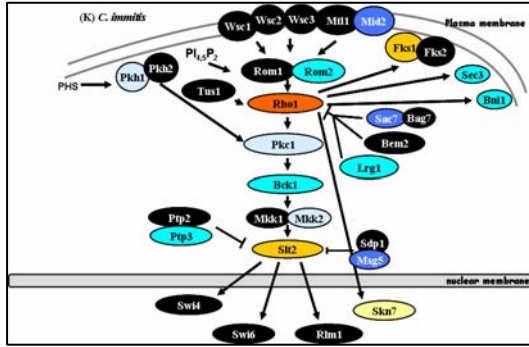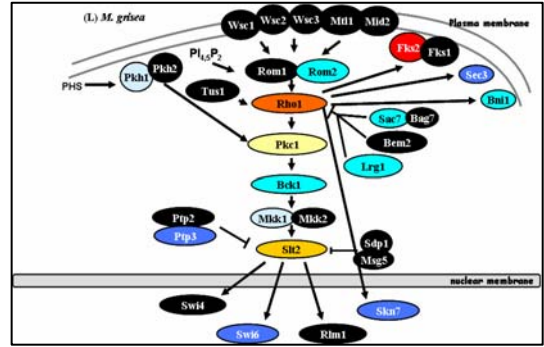

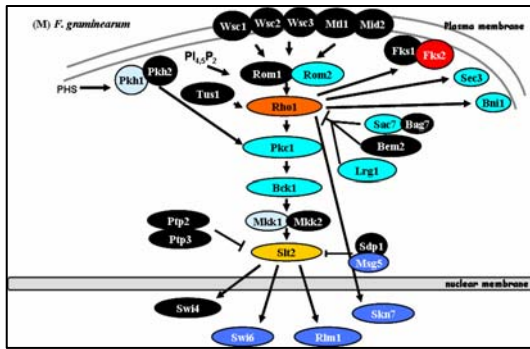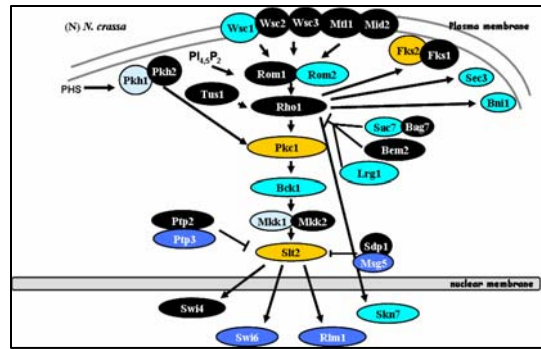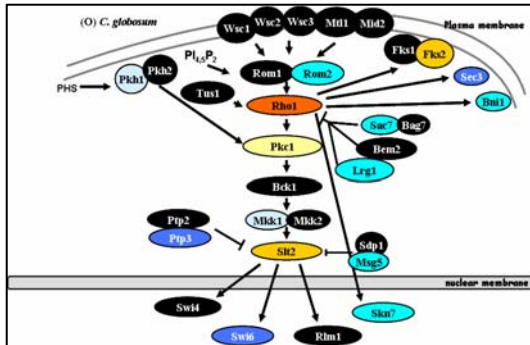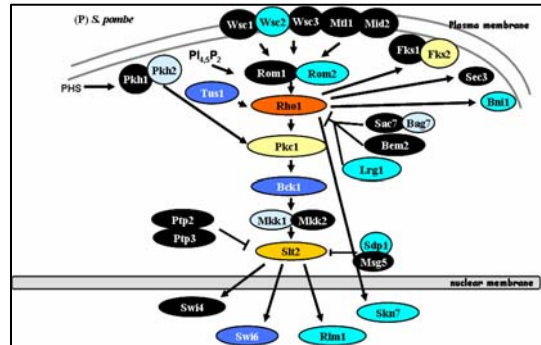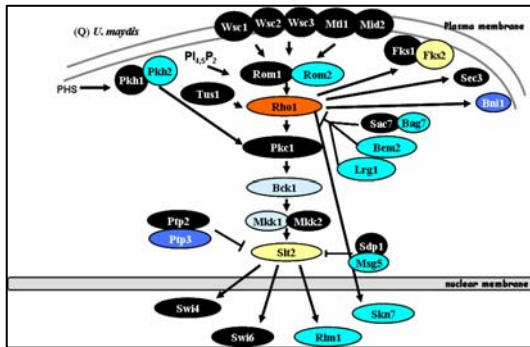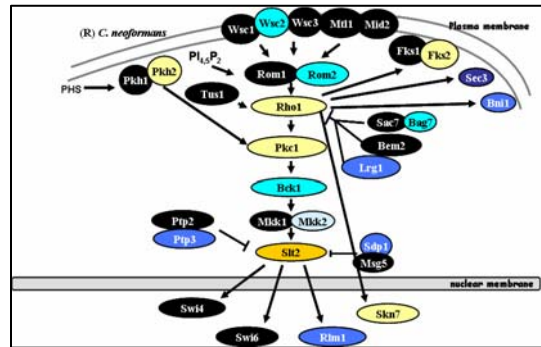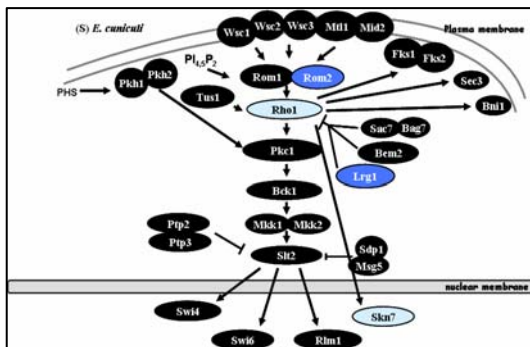

Supplement: Additional file 3 — Conservation of fungal osmotic, oxidative and cell wall stress pathways. Figures illustrating the degree of conservation of signalling molecules on stress pathways in each of the fungal species examined: (A) osmotic stress signalling pathway; (B) oxidative stress signalling pathway; (C) cell wall cell stress signalling pathway. [file 1471-2148-9-44-S3.pdf]
