# Supplementary material for: Phylogenetic diversity of stress signalling pathways in fungi
Source: BMC Evol Biol. 2009 Feb 21;9:44. doi: 10.1186/1471-2148-9-44 (PMC2666651; doi:10.1186/1471-2148-9-44)

# Additional file 5

A plot showing that no significant correlation exists between stress pathway conservation and oxidative stress responses. The mean % identity for proteins of the oxidative stress pathway relative to their *S.cerevisiae* orthologues, was plotted against the absolute difference between *S.cerevisiae* growth inhibition and that of comparator species when treated with either hydrogen peroxide (diamonds) or menadione (squares).


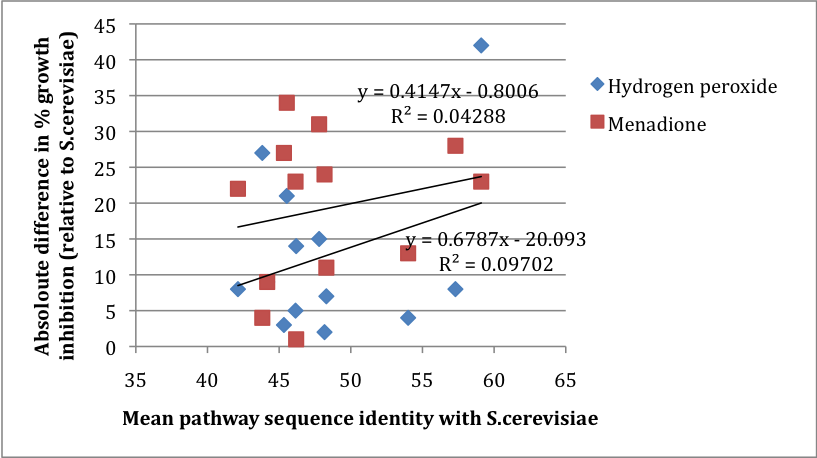

Supplement: Additional file 5 — Proteins used for phylogenetic analysis. Details of fungal orthologues used to construc the phylogenetic tree. [file 1471-2148-9-44-S5.doc]
